# Population-based analysis of the number of thrombectomies performed after cerebral ischemic stroke and prognostic factors of mortality in France

**DOI:** 10.1007/s10654-023-01074-5

**Published:** 2024-01-14

**Authors:** Fabien de Oliveira, Lucas Léger, Vincent Costalat, Ihssen Belhadj, Maxime Pastor, Héléne de Forges, Jean-Paul Beregi, Thierry Boudemaghe, Julien Frandon

**Affiliations:** 1 Department of Medical Imaging, IMAGINE UR UM 103, IPI platform, Montpellier University, Nîmes University Hospital, place Prof Robert Debré, 30029 Nîmes Cedex 9, France; 2 UA11 INSERM - UM Institut Desbrest d Épidémiologie et de Santé Publique (IDESP), Montpellier, France; 3 Department of Medical Information, Methods and Research, Centre Hospitalier Universitaire de Nîmes, University of Montpellier, Nîmes, France; 4 Neuroradiology, Gui de Chauliac Hospital, Montpellier University Medical Center, Montpellier, France

## Introduction

Ischemic stroke management with occlusion of large blood vessels in the anterior circulation has been ground broken with the development of mechanical thrombectomy (MT). Indeed, in multiple randomized trials, this new treatment has shown greater efficacy than thrombolysis alone in the first 6 h following a stroke [1–3]. Due to its relative novelty, health systems and hospitals worldwide have had to adapt their care offer to provide for this treatment.

Studying the exact number of MTs performed in the whole territory and per region will make it possible to identify territories with very low access to thrombectomy, improve the regional network and homogenize the available care.

The purpose of this study was to evaluate the nationwide utilization level of MTs in France through a population-based analysis over a two-year period (2018–2019). Secondary objectives were to evaluate mortality following MT, and identify predictive factors.

## Patients and methods

### Study design

This retrospective study analyzed data extracted from the standard French national hospital discharge database, known as the PMSI (*Programme de Médicalisation des Systèmes d’Information*), [4] over the 2018–2019 period (Declaration number 2,203,389 v.0).

### Data selection

In France, the PMSI database records all procedures performed during all hospitalizations, including daycare or longer, in private or public institutes [4]. Since July 2017, MT has become a well-defined, specific procedure coded as EAJF341 under the common classification of medical acts (*Classification commune des actes médicaux*, CCAM) and is thus coded as such in the PMSI database. To study two complete years, all data covering 2018 and 2019 were extracted. Data on patients aged 20 or over with “stroke” as the main diagnosis (International Classification of Diseases code 10 : I63.0) associated or not with MT were extracted for inclusion in the study. The code specific to thrombectomy was then checked. Both initial and recurrent strokes – apart from early recurrences, i.e. recurrences occurring during the ongoing hospital treatment phase for a first incidence - were included.

### Endpoints and assessments

The primary endpoint was the number of MTs performed across the territory, standardized per age, sex and percentage of strokes. Secondary endpoints were the mortality rate at 1 year after thrombectomy, and associated risk factors.

Depending on the number of MTs performed in 2018 and 2019, and according to the presumed experience of the operators, stroke centers were classified as follows [5] :

- very small MT centers with very few thrombectomies performed (1 to 9 MTs/year): no experience.
- small MT centers with few thrombectomies performed (10 to 49 MTs/year): low experience.
- occasional MT centers with 50 to 99 MTs/year (> 1/week): regular experience.
- regular MT centers with 100 to 199 MTs/year, (2 to 4/week): good experience.
- high-volume MT centers with 200 to 399 MTs/year (more than 4/week): very good experience.
- very high-volume centers with more than 399 MTs/year (> 1/day): experts.

Endpoints concerning mortality were death rates during hospital stay and at 1 year. Data concerning comorbidities including chronic heart, respiratory or renal failure, cancer, diabetes mellitus and cardiovascular pathologies were also extracted from the PSMI to assess prognostic factors. The study on mortality only focused on patients who had undergone thrombectomy.

### Statistical analysis

Statistical analyses were carried out using R software (version 4.2.2).

Standardized prevalence was calculated with the number of MTs for 100 000 inhabitants per year and per department, with standardization according to sex and age. The reference population was defined with the number of inhabitants in France over the period.

A generalized linear mixed model was used to estimate the one-year probability of death for patients undergoing thrombectomy (death during hospital stay or subsequent visits). To account for potential differences in mortality between centers, centers were treated as random effects. All variables characterizing patients and their hospital stays: length of stay, age, gender, Charlson comorbidity index, the 17 components of the Charlson index, center size (in terms of the number of thrombectomies performed), month, and year were treated as fixed effects. The linearity of quantitative variables were assessed and transformed to achieve a linear effect. The natural logarithm of the length of stay and the square root of the number of thrombectomies were thus taken. The final multivariate model included the variables of interest in the study, as well any variables that emerged as being significant through the likelihood-ratio test.

## Results

### Patient characteristics

Over the study period, 231,947 strokes were reported in the PSMI with 115,451 in 2018 and 116,496 in 2019, i.e. an increase of 0.9%. For the same time period, 12,817 MTs were reported with 5,971 in 2018, i.e. 5.2% of all strokes and 6,846 in 2019, i.e. 5.9% of all strokes, corresponding to a 14.7% increase in MTs. Patient characteristics were similar between patients who had undergone strokes and those treated with MT. Patients were 6,372 men (49.7%) and 6,445 women (50.3%). The median age was 76.0 years [IQR: 65–85] years for patients with stroke and 73.0 years [IQR: 62–82] for patients treated by MT.

### Distribution of MTs

Data were analyzed for 101 departments with a total population of 50.7 million inhabitants in 2018 and 51.0 million in 2019 (aged 20 or over). Between 2018 and 2019, the number of MTs performed increased from 11.8 to 13.4 per 100,000 inhabitants. The mean number of MTs was 12.61 per 100,000 inhabitants over the study period, ranging from 0.32 (Guyane, a French overseas territory) to 20.04 (Paris). Overall, 53% of departments, without a regular or higher-volume MT center (> 100 MTs/year), reported an MT rate/100,000 inhabitants below the national average (+/-10%) (Figure).

Regular MT centers or above accounted for 80.6% of all MTs performed in 2018 and 85.3% in 2019. Between 2018 and 2019, the number of MTs performed rose by 738 in regular centers, whereas it only rose by 36 for very high-volume centers. Small centers (< 50 MTs/year) accounted for 5.9% of all MTs performed during the study period (Table 1).

### Mortality rate and associated factors

Of the 12,817 patients who underwent MTs in 2018 and 2019, 2,807 (21.9%) patients died during the following year. In the multivariate analysis, independent factors found to be significantly associated with mortality were age, length of hospital stay, cancer, chronic heart, renal or pulmonary failure, a history of cardiovascular disease and diabetes mellitus (Table 2). Regarding age, the increase in the risk of mortality was linear, with an inflection point and sharper rise after 75 years. Regarding duration of hospitalization, the variation followed a polynomial pattern with an initial peak during the first few days, followed by a linear increase. Female gender was correlated with a better prognosis after MT. There was no correlation between the center’s experience and mortality rate at 1 year.

**Fig. 1:**
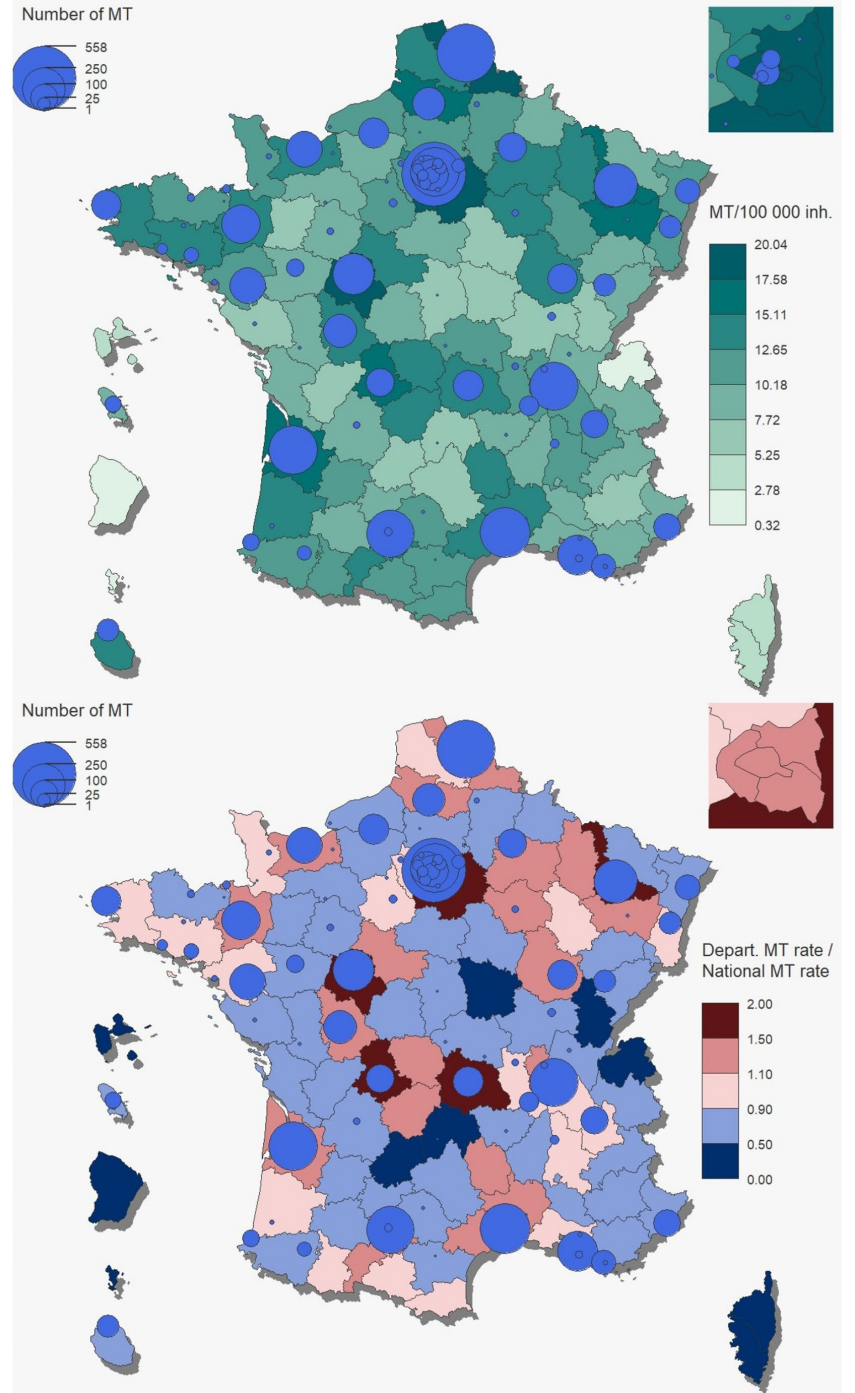
Distribution of mechanical thrombectomies (MTs) in France (mainland and overseas) during the study period (2018–2019). Top left: the number of MTs performed for 100 000 inhabitants (green scale) and, bottom left: the number of MTs performed per department compared to the national average number of MTs (red and blue scale). The blue circles represent the number of MTs performed in MT centers. *MT rates were normalized according to the department/national population. Departments performing more MTs than the national average number of MTs are shown in light and dark red and those that perform fewer MTs are shown in light and dark blue*

**Table 1:** Number of strokes and mechanical thrombectomies (MTs) per MT center group size in 2018 and 2019, and their evolution from 2018 to 2019. Only MT centers performing more than 10 MTs in the year are presented

	2018			2019			Evolution	
**MT center group size**	**Number of strokes**	**Number of MTs**	**Strokes treated by MT (%)**	**Number of strokes**	**Number of MTs**	**Strokes treated by MT (%)**	**Number of MTs**	**Strokes treated by MT (%)**
Overall	115,451	5,971	5.17	116,496	6,846	5.88	875	0.71
> 400	8278	995	12.0	7730	1031	13.3	36	1.3
200–399	13,032	2495	19.1	13,996	2748	19.6	253	0.5
100–199	8355	1193	14.3	12,565	1931	15.4	738	1.1
50–99	9158	800	8.7	6886	606	8.8	-194	0.1
10–49	9912	356	3.6	10,781	400	3.7	44	0.1

**Table 2:** Multivariate analysis of predictive factors for mortality following treatment by mechanical thrombectomy

Variable	Variable type and effect	Effect	OR	LRT	p
Length of stay (logarithm)	Continuous (polynomial of degree 6)	Complex (maximum probability of death on Day 1, followed by a decrease to a minimum probability on Day 15, and a gradual, constant increase thereafter)	N/A	1288.06	< 0.0001
Age	Continuous (polynomial of degree 3)	Steady increase in risk up to age 75, followed by a sharp increase in risk beyond that age	N/A	505.03	< 0.0001
Chronic heart failure	Binary	Increases the risk	1.75 (1.52-2.00)	62.52	< 0.0001
Sex	Binary	Lower risk in women	0.72 (0.66–0.80)	41.33	< 0.0001
Cancer	Binary	Increases the risk	1.78 (1.44–2.20)	27.20	< 0.0001
Metastatic cancer	Binary	Increases the risk	2.03 (1.41–2.92)	14.42	< 0.0001
Diabetes mellitus	Binary	Increases the risk	1.33 (1.15–1.54)	14.18	0.00017
Chronic renal failure	Binary	Increases the risk	1.40 (1.14–1.71)	10.52	0.00118
Chronic pulmonary failure	Binary	Increases the risk	1.40 (1.13–1.75)	8.89	0.00287
History of cardiovascular disease	Binary	Increases the risk	1.26 (1.05–1.51)	5.92	0.01493
Number of MTs (square root)	Continuous (linear)	No effect	N/A	0.84	0.35837

## Discussion

The results of this nationwide study demonstrate the feasibility of a precise survey on mechanical thrombectomy treatment in France by analyzing the number of thrombectomies performed, per stroke center and per department, based on data extracted from the PMSI database. The number of thrombectomies had increased by 14.7% over the 2-year study period, mainly in regular MT centers, with a 1% increase in the number of strokes at the same time. Our results show an inequality in the use of thrombectomy throughout the territory. The 1-year mortality rate after thrombectomy was high (21.9%) but unrelated to the number of MTs performed per center.

Our analysis shows that centers doing over 50 thrombectomies per year account for 94% of all thrombectomies performed. This distribution is different from what was found in a previous study in Germany in 2016, with only 80% of thrombectomies performed by same size centers and a higher number of MT performed with 10,692 patients treated [6]. These are raw data that need to be adapted to age and gender, but they argue for a less centralized organization for greater efficiency. However, the analysis per center-size shows that most of the increase in MTs performed in the country was due to regular and not very high-volume MT centers. Regular MT centers must be developed to improve access to MT throughout the territory.

The mortality rate was consistent with results from previously published studies, with mortality rates of 14 to 21% in the first 3 months following thrombectomy [7, 8]. We found no difference in mortality at 1 year post thrombectomy between the different-sized centers performing thrombectomy. In France, MTs can be performed either by neurointerventional physicians or by more general interventional physicians, depending on the center. Other studies have already found that the performances of interventional radiologists did not significantly differ from those of neurointerventional physicians in performing thrombectomies [9]. More interventional radiologists should be trained in MT to improve the available care.

This PMSI database has certain limitations. The data may include unusable records containing errors, surrogate data (e.g. the generic location code for an unknown patient location) or missing data, especially with an emergency mention. However, as hospitals use the PMSI for the billing process they are extremely vigilant about correct encoding. The PMSI only codes information on hospital stays. Death occurring outside the hospital, clinic or rehabilitation structure are not recorded, which may have led to an underestimation. Furthermore, although stroke is a major cause of disability, stroke impairment could not be evaluated.

In conclusion, this study shows the feasibility of a precise survey of MT in France, per stroke center and per department based on PMSI data analysis. It could be used to benchmark centers, follow activity and create a national MT observatory. This study argues for decentralized care with the development of medium-sized centers to improve the use of thrombectomies without risking an excess of mortality compared to highly experienced centers.

## Data Availability

All data to support the findings of this study are available from the authors upon reasonable request.

## References

[1] Berkhemer OA, Fransen PSS, Beumer D, van den Berg LA, Lingsma HF, Yoo AJ (2015). A randomized trial of intraarterial treatment for acute ischemic Stroke. N Engl J Med.

[2] Campbell BCV, Mitchell PJ, Kleinig TJ, Dewey HM, Churilov L, Yassi N (2015). Endovascular therapy for ischemic Stroke with perfusion-imaging selection. N Engl J Med.

[3] Goyal M, Demchuk AM, Menon BK, Eesa M, Rempel JL, Thornton J (2015). Randomized assessment of rapid endovascular treatment of ischemic Stroke. N Engl J Med.

[4] Boudemaghe T, Belhadj I. Int J Epidemiol. 2017;46:392–392d. Data Resource Profile: The French National Uniform Hospital Discharge Data Set Database (PMSI). 10.1093/ije/dyw359 28168290

[5] Krogias C, Bartig D, Kitzrow M, Brassel F, Busch EW, Nolden-Koch M (2017). [Availability of mechanical thrombectomy for acute Stroke: analysis of the health care reality in Germany]. Nervenarzt.

[6] Weber R, Eyding J, Kitzrow M, Bartig D, Weimar C, Hacke W (2019). Distribution and evolution of acute interventional ischemic Stroke treatment in Germany from 2010 to 2016. Neurol Res Pract.

[7] Junttola U, Lahtinen S, Isokangas J-M, Hietanen S, Vakkala M, Kaakinen T et al. Long-term mortality after endovascular thrombectomy for stroke. J Stroke Cerebrovasc Dis [Internet]. Elsevier; 2022 [cited 2023 Jan 9];31. Available from: https://www.strokejournal.org/article/S1052-3057(22)00526-2/fulltext. 10.1016/j.jstrokecerebrovasdis.2022.106832 36257143

[8] Li X, Li C, Zhou J, Liu A, Zhang Y, Zhang A (2022). Predictors of ninety-day mortality following mechanical thrombectomy for acute large vessel occlusion Stroke. Clin Neurol Neurosurg.

[9] Sacks D, Dhand S, Hegg R, Hirsch K, McCollom V, Sarin S (2022). Outcomes of Stroke Thrombectomy performed by Interventional Radiologists versus Neurointerventional Physicians. J Vasc Interv Radiol JVIR.

